# Early‐Life Climbing Stratifies the Metabolome and Mortality Risk in Genetically Identical Flies

**DOI:** 10.1111/acel.70299

**Published:** 2025-11-26

**Authors:** Benjamin R. Harrison, Yangxi Sun, Tom Nonacs, Harini Shankar, Danijel Djukovic, Daniel Raftery, Daniel E. L. Promislow

**Affiliations:** ^1^ Department of Anesthesiology and Pain Medicine, Northwest Metabolomics Research Center University of Washington Seattle Washington USA; ^2^ Department of Lab Medicine and Pathology University of Washington Seattle Washington USA; ^3^ Barnard College New York New York USA; ^4^ Jean Mayer USDA Human Nutrition Research Center on Aging Tufts University Boston Massachusetts USA

**Keywords:** covariance networks, isogenic variation, metabolomics, mortality

## Abstract

Studies in laboratory organisms typically minimize all environmental and genetic variation other than the intervention of interest. In aging studies, these highly controlled conditions have yielded profound insights into aging. But even within isogenic cohorts of lab animals in controlled environments, we observe substantial variation in lifespan. Here we exploited the climbing behavior of *Drosophila* to study variation in mortality among isogenic populations in a controlled environment. We show that fractionating large cohorts of relatively young isogenic flies by climbing behavior predicts future mortality risk and stress sensitivity. Using metabolomics to dissect this variation, we found metabolites whose abundances differ among the fractions. We also took advantage of the large number of individuals in each fraction, and the ease with which they can be collected, to explore the covariance structure of metabolites in flies that are genetically identical, but divisible into short‐lived and long‐lived fractions. In doing so, we identified metabolites and metabolic pathways as candidate biomarkers of intrinsic mortality risk.

## Graphical abstract

Isogenic *Drosophila melanogaster* vary greatly in longevity. Fractionation of an isogenic cohort based on climbing behavior early in life stratifies variation in the metabolome, longevity and mortality risk offering a tractable system to study intrinsic sources of variation in aging.

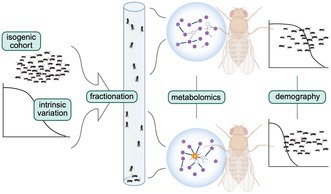

## 1 Introduction

For over a century, quantitative genetic studies of aging have estimated variances due to genetic (*V*_G_) and environmental (*V*_E_) factors that contribute to age‐related mortality. We can further parse *V*_E_ into variation due to the micro‐environment specific to each individual (*V*_ES_), and that due to common (or “general”) environmental variation shared among individuals (*V*_EC_), such that *V*_E_ = *V*_EC_ + *V*_ES_ (Falconer and Mackay 1996). Sources of *V*_ES_ can include heterogeneity in intrinsic factors such as gene expression, development, and behavior (Kirkwood et al. 2005). The extent of *V*_ES_ for lifespan is considerable (Khazaeli et al. 1998; Kirkwood et al. 2005). For example, under controlled environments, isogenic flies, nematodes, or rodents exhibit a coefficient of variation in lifespan of at least 20% (Gärtner 2012; Khazaeli et al. 1998; Kinser et al. 2021). While the extent of *V*_ES_ for human lifespan is perhaps unmeasurable, estimates of V_G_ typically fall between 10% and 30%, leaving substantial *V*_E_ (Ruby et al. 2018; van den Berg et al. 2017).

Studying isogenic cohorts in controlled environments limits the extrinsic sources of variance such that the intrinsic component of *V*_E_ is most exposed (Khazaeli et al. 1998). Investigations of intrinsic mortality in yeast (Janssens and Veenhoff 2016), nematodes (Oswal et al. 2022; Rea et al. 2005), and flies (Carey et al. 2006; Rera et al. 2012; Tower 2023) have identified biomarkers of mortality that manifest within isogenic cohorts including variation in physiological traits, behavior, and gene expression. In *Drosophila*, locomotion, gut integrity, and fecundity each associate with age and longevity (Carey et al. 2006; Rera et al. 2012; Zhao et al. 2022). The vertical climbing behavior of *Drosophila* is associated with both age and longevity differences among fly strains, and has been used as a model of physiological decline (Jones and Grotewiel 2011; Rhodenizer et al. 2008), particularly for its similarity to the decline in human walking speed late in life and the strength of human walking speed as a biomarker of mortality risk (Jones and Grotewiel 2011; Studenski et al. 2011).

As early as 10 days of age, gut integrity in *Drosophila* predicts longevity (Rera et al. 2012), and associates with variation in the transcriptome (Zane et al. 2023), and microbiome (Clark et al. 2015), clearly indicating that we might be able to use variation in endophenotypes as biomarkers of mortality risk. Among endophenotypes, the metabolome is comprised of the small molecules that make up the structural and functional building blocks of the cell. Age‐related traits in *Drosophila* are well reflected in metabolomic variation (Avanesov et al. 2014; Wang et al. 2022; Zhao et al. 2022), and intervention studies in *Drosophila* have demonstrated causal connections between metabolites and lifespan (Kabil et al. 2011; Obata and Miura 2015; Parkhitko et al. 2016, 2021; Su et al. 2019). In addition to identifying metabolites whose abundance associates with age or mortality, covariance within the metabolome may also give insight into the aging process (Steuer et al. 2003), pointing to activity in specific metabolic pathways (Krumsiek et al. 2011).

Here we targeted biomarkers of intrinsic mortality risk in *Drosophila*. We exploited variation in climbing behavior to fractionate large numbers of isogenic flies in a controlled environment. These fractions differed in mortality risk and stress sensitivity, establishing these sub‐populations as a model of intrinsic mortality risk. By sampling flies among the fractions, we found several metabolites, including those enriched in the tryptophan, fatty acid, and pantothenate metabolism pathways, whose abundance was associated with climbing fractions. We found pairs of metabolites whose covariance patterns varied among different climbing fractions, in which network analysis indicated that taurine and hypotaurine metabolism may play a central role in metabolite covariation differences among isogenic flies.

## 2 Results

### 2.1 Climbing Behavior Associates With Mortality Risk

To test the hypothesis that the climbing behavior of *Drosophila* predicts mortality risk, we measured relative climbing in replicates of approximately 200 flies each that were sampled from a cohort of ~3300 age‐matched mated female *Drosophila* of the lab strain Canton‐S. Flies in these samples were then fractionated based on relative climbing behavior (Section 2, Figure S1). Four replicates were fractionated from the cohort at 4 weeks of age (“Week 4,” fly‐age 29 ± 1 day), when 92.3% of flies were still alive, and seven replicates were fractionated at 6 weeks of age (“Week 6,” 43 ± 1 day), when 79.3% of flies were alive (Figure S1). For Week 4 and Week 6 flies, we separated fractions based on their climbing performance (the number of trials flies were in the top group). This led to five separate groups including those that climbed to the top in 3 of 3 trials (“top”), 2 of 3 trials (“mid‐top”), 1 of 2 trials (“middle”), 1 of 3 trials (“mid‐bottom”), and 0 of 3 trials (“bottom”). This procedure exploits the *relative* climbing of flies and does not require flies to climb at a particular velocity, or even to climb at all. Instead, flies in the upper fraction are merely those that have climbed above the level where we introduce a partition, at the time when we deemed approximately half of the flies to be located above that level in the chamber (Section 2, Figure S1). After the fractionation procedure, each fraction of flies was maintained in replicate vials to track survivorship.

**FIGURE 1:**
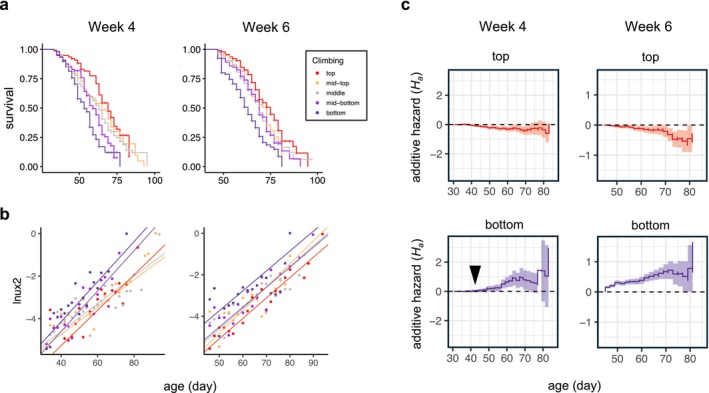
Climbing behavior predicts mortality risk. (a) Kaplan–Meier survivorship for flies fractionated at either Week 4 (Day 29 ± 1), or at Week 6 (43 ± 1), *n* = 78–439 flies per fraction at each age. Vertical ticks along plot lines indicate censored flies. (b) The log mortality rate (ln(*μ*(*x*))) versus age for flies in each climbing fraction. Lines show Gompertz models fit by maximum likelihood. (c) The additive hazard (*H*_a_) and its 95% pointwise confidence intervals (shaded borders) fit by Aalen regression to data from the bottom fraction (purple), or the top fraction (red), using the hazard of the middle climbing fraction as the reference population. The additive hazard in the bottom fraction, when fractioned at either Week 4 or Week 6, is greater than the reference population (*H*_a_ > 0, *p* < 8.32 × 10^−4^), whereas flies in the top fraction from either Week 4 or Week 6 have reduced hazard (*H*_a_ < 0, *p* < 0.015). Note the additional hazard in the bottom fraction from Week 4 shows an ~10 days post‐fractionation onset (arrow in c) compared to the immediate risk in the bottom fraction from Week 6. Aalen regression for all climbing fractions is shown in Figure S2, and summary statistics are given in Table S1.

We then asked if mortality risk differed among the fractions. A Kaplan–Meier estimate showed that climbing propensity was associated with higher survivorship (Figure 1a). To compare mortality among the five climbing fractions, we fitted the Gompertz mortality curve (Equation 1),

(1) $$\mu \left(x\right)={\mathrm{\alpha e}}^{\mathrm{\beta x}}$$

Taking the log of both sides,

(2) $$\ln\left(\mu \left(x\right)\right)=\ln\left(\alpha \right)+\mathrm{\beta x}$$

where *μ*(*x*) is the intrinsic mortality rate at age *x*, *α* is the age‐independent term (the intercept on a plot of log‐mortality versus age), and *β* is the slope of the line, and is typically interpreted as the rate of aging. After fractionation at Week 4, both *α* and *β* associated with climbing [likelihood ratio test (LRT), *p* = 5.3 × 10^−9^, and *p* = 5.6 × 10^−4^, respectively, Figure 1b], with the poorest climbing fractions showing increased intrinsic mortality and higher rate of increase compared to the other fractions. For flies fractionated at Week 6, intrinsic mortality differed among the fractions, with the poorest climbers showing increased risk. However, the rate of aging (*β*) did not differ among the fractions (LRT, *p* > 0.05, Figure 1b). To examine age‐dependent risk in each fraction, we fitted a time‐dependent hazard model to each cohort by Aalen regression, using the flies in the “middle” climbing group as a reference population. When fractioned at either Week 4 or Week 6, top flies had significantly reduced cumulative additive hazard (*H*_a_ < −4.8 × 10^−4^, *p* ≤ 0.014, Section 2), whereas bottom flies showed greater hazard (*H*_a_ > 1.46 × 10^−3^, *p* ≤ 8.32 × 10^−4^, Figure 1c, Figure S2). Consistent with the significantly higher Gompertz *β* among the Week 4 survivorship, *H*_a_ in the poorest climbing flies did not initially deviate from the reference population but did so approximately 10 days following fractionation (Figure 1c).

**FIGURE 2:**
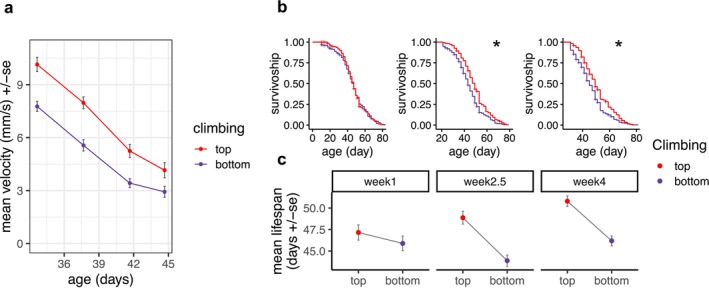
The behavioral difference between climbing fractions persists after fractionation and manifests in early adulthood. (a) Mean velocities of the 49–294 flies in the bottom and top fractions that were climbing in the demography vials following fractionation at Week 4. The top and bottom fractions differ significantly in age‐independent velocity (Section 2, *p* = 1.61 × 10^−5^), and there is no difference between fractions in the rate of decline in velocity with age (*p* = 0.25). (b) Kaplan–Meier survivorship, and (c) restricted‐mean lifespan of the top and bottom climbing fractions of flies fractionated at Week 1 (Day 8 ± 1), Week 2.5 (Day 19 ± 1) and Week 4 (Day 29 ± 1), with error bars indicating ±standard error of the mean. Asterisks indicate significantly different survival among top and bottom climbing groups within each trial (log‐rank *p* < 0.05). Summary statistics are given in Table S1.

We tested for differences in climbing velocity among the top and bottom fractions, immediately following fractionation and several days afterward. Flies from the top fractions climbed consistently faster than those in the bottom fraction, and both populations slowed with age (Figure 2a). We then tested the hypothesis that latent variation in climbing behavior and mortality is due to variation that manifests prior to Week 4 in adulthood. A cohort of > 1300 flies was raised in the same manner as before and subsets of this cohort were fractionated at Week 1 (8 ± 1 day), Week 2.5 (19 ± 1 day) or Week 4 (29 ± 1 day) of age. We repeated the same fractionation procedure as before, but only measured survival among the top and bottom fractions. We were able to reproduce the effect of Week 4 fractionation on mortality, with the top fraction outliving the bottom fraction (mean lifespan of 50.3 days vs. 45.6 days, log rank *p <* 10^−4^). This was also true for the flies separated at Week 2.5 (*p <* 10^−4^), but not at Week 1 (*p =* 0.92, Figure 2).

### 2.2 Stress Resistance

**FIGURE 3:**
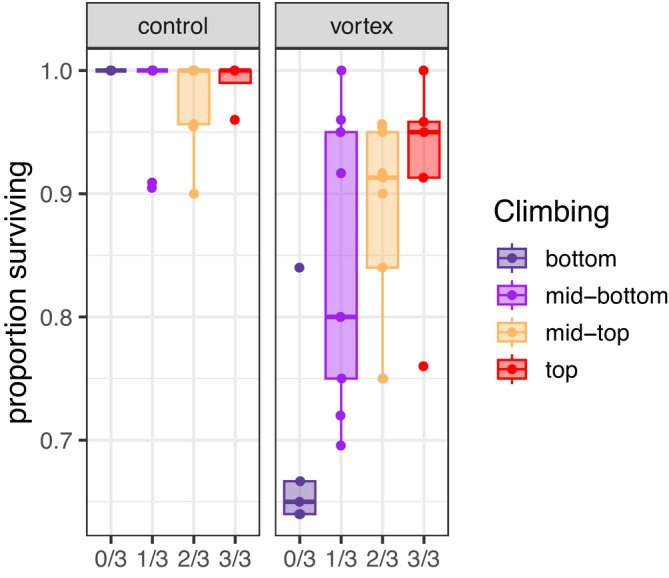
Climbing fractions associate with stress sensitivity. Four‐week‐old flies were fractionated into climbing groups according to the main text (*n* = 9 replicates), and then subjected to a bang assay, with 90 s of traumatic stress on a mechanical vortex mixer (vortex, Section 2). The proportion of (*n* = 64–116) flies alive 24 h after trauma was strongly associated with climbing fraction (Section 2, likelihood ratio test, *p* = 6.6 × 10^−8^). Summary statistics are given in Table S1.

We then asked if climbing fractions were associated with sensitivity to traumatic stress. To assay for traumatic stress sensitivity, a cohort was raised as before. At Week 4, nine replicate fractionations were performed, followed immediately by bang assays on the top and bottom fractions (Section 2). While post‐trauma survival of flies from the top fraction averaged 92% (*n* = 5 vials), an average of only 69% of flies in the bottom fraction survived trauma, with survivorship dropping monotonically by climbing fraction (LRT, *p =* 6.6 × 10^−8^, Figure 3).

### 2.3 Climbing and Mortality Associates With the Metabolome

**FIGURE 4:**
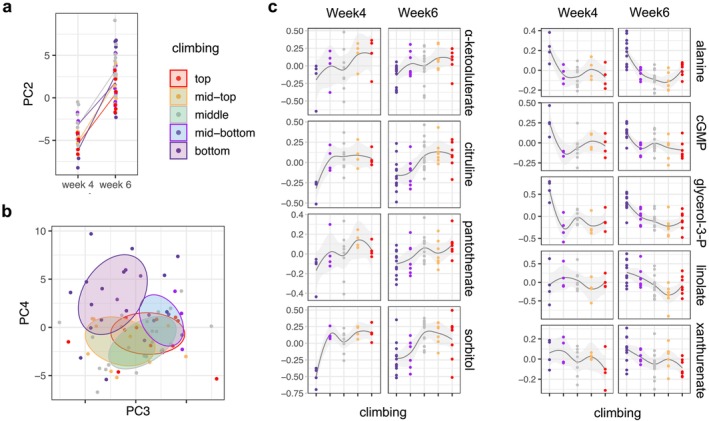
Latent variation in the metabolome. (a) Principal component two (PC2) strongly separates flies by age (left panel, lines connect the mean PC2 of each climbing fraction). (b) PC3 and PC4 separate flies primarily among the poorer‐climbing “bottom” fraction. (c) Ordinal regression of climbing fraction on metabolite level at either Week 4 or Week 6 identified nine metabolites affecting the log odds of ranked climbing fraction (Section 2, FDR ≤ 0.05); left and right panels show metabolites whose increasing abundance predicts either higher climbing, or lower climbing, respectively.

We used targeted metabolomics to identify biomarkers for climbing ability and survival. Replicate samples of flies were collected for metabolomics from the initial experiment immediately after fractionation while flies were being dispersed back into vials for demography (Figure 1). This gave four to five samples for each climbing fraction at Week 4, and seven to eight samples per fraction at Week 6. We measured 160 aqueous metabolites in each sample by liquid chromatography‐mass spectrometry (LC–MS, Section 2). Together, the first four principal components (PCs) accounted for 39.7% of the total variation, with components highly associated with fly age (PC_2_, ANOVA *F*_1, 52_ = 187.0, *p <* 2.2 × 10^−16^), and with climbing fraction (PC_3_ and PC_4_, ANOVA *F*_4, 52_ = 3.70, *p =* 0.01 and *F*_4, 52_ = 7.09, *p =* 1.2 × 10^−4^, respectively) (Figure 4 and Figure S3).

To identify metabolites associated with climbing, we treated climbing fraction as five ordinal levels, from bottom to top, and asked if there were metabolites associated with the odds of being in each climbing fraction, versus a higher fraction (Section 2). These odds were associated with lower levels of five metabolites (glycerol‐3‐phosphate, alanine, cyclic‐GMP, xanthurenate, and linolate) and higher levels of four metabolites (sorbitol, citrulline, α‐ketogluterate, and pantothenate) (FDR < 0.05, Figure 4c), with common effects in flies sampled at Week 4 and at Week 6 (ANOVA *p* > 0.05). We then asked if any biological processes or pathways were enriched by these nine metabolites, using a network diffusion‐based approach (Section 2) (Picart‐Armada et al. 2018). We found enrichment for two pathways—biosynthesis of fatty acids (dme01040), and tryptophan metabolism (dme00380)—as well as the KEGG module, pantothenate biosynthesis (M0019), a short pathway wherein pantothenate is generated from valine and aspartate (FDR < 0.05).

### 2.4 Latent Metabolome Co‐Variation

We next identified pairs of metabolites whose covariance was associated with climbing. Given *N* = 160 metabolites, there are *N* (*N* − 1)/2 = 12,720 potentially covarying pairs. Within this set of possible pairs, we found 1966 whose abundance covaried among the 71 fly samples (Pearson's correlation, FDR < 0.05, Section 2). Among this set, we found 103 pairs whose pattern of covariance was associated with climbing behavior (FDR < 0.05, Section 2, Figure S4), such that covariation for a given pair differed along the ordinal axis of climbing fraction. Notably, the metabolite pairs were not necessarily correlated in all climbing fractions. For example, the levels of taurine and methylthioadenosine are positively correlated in the top two fractions (*p* < 0.03) and not correlated among the intermediate or bottom fractions (*p* > 0.05, Figure S4).

We next sought to determine if the 103 out of 1966 pairs that were associated with climbing behavior were more than one would expect by chance. To test this, we compared the number of pairs expected from randomized data. In particular, by permuting the climbing fraction across all samples, preserving the within‐sample metabolite covariance, we asked how many pairs of metabolites associate with randomized ordinal climbing fractions. In 1000 permutations, we found an average of 17.0 such pairs in contrast to the 103 we observed (empirical *p* = 0.011, Figure S4).

**FIGURE 5:**
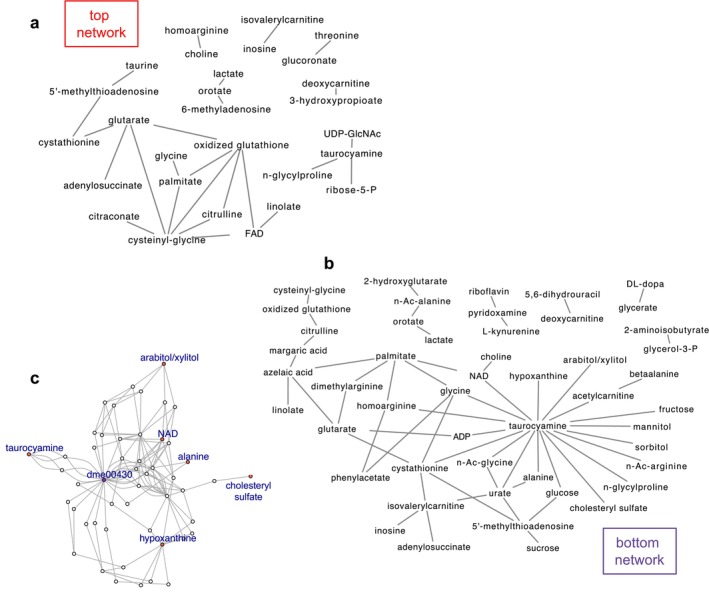
Latent covariation in the metabolome. Network plot of metabolite pairs among the top climbing fraction (a) or the bottom climbing fraction (b). (c) A sub‐network within the KEGG network that contains paths connecting five of the metabolites that covary in the bottom network (red nodes), and which all contain the node dme00430 (taurine and hypotaurine metabolism, purple node).

To explore the metabolome covariance in flies of the top and bottom fractions specifically, we examined the 55 metabolite pairs that were at least nominally correlated (*p* ≤ 0.05), in the bottom climbing fraction, and the 25 pairs in the top climbing fraction. Nineteen of these pairs covaried among flies in both the top and bottom fractions, albeit to different degrees (Figure S4). We then examined the network structure among metabolites covarying in the top and bottom fractions by constructing edges (i.e., connections) between metabolite pairs. The network among the bottom fraction shows more connectivity than the network of the top fraction and among its nodes, taurocyamine is highly connected, with edges to 18 other metabolites (Figure 5).

### 2.5 Taurine and Hypotaurine Metabolism Node Connects Covarying Metabolites in Poor‐Climbing Flies

We then explored the relationship among these metabolites in the *D. melanogaster* KEGG network. Unlike the metabolite covariance networks, which only contain nodes corresponding to metabolites, the KEGG network contains additional nodes that correspond to reactions and enzymes, as well as individual nodes that represent biological pathways and short biochemical processes (modules). Among the nodes in the specific KEGG network that we analyzed are 133 pathways, 174 modules, 768 enzymes, 5694 reactions, and 4162 metabolites, including 133 of the targeted metabolites that we measure (Section 2), and a total of 31,842 edges. Among these 31,842 edges, none directly connect two metabolites, but rather metabolites in KEGG are connected to each other by reactions, enzymes, and often by pathways or modules. Metabolites are connected in the KEGG network based on their known biochemical reactions and membership within pathways, and so we reasoned that the nodes along the paths connecting metabolites could indicate the biological processes by which they relate (Krumsiek et al. 2011). Here we test this reasoning by asking if pairs of covarying metabolites are closer to each other in KEGG than two randomly selected metabolites, and then if there are biological pathways enriched among the paths that connect covarying metabolites in either the poor‐climbing, or in climbing‐prone flies.

We found that covarying pairs of metabolites tended to be closer to each other in KEGG than expected by chance (Figure S5, Section 2). This was true when comparing the paths connecting pairs whose covariance met a fixed FDR threshold of 0.05, versus all possible pairs (*χ* ^2^ = 28.9, df = 4, *p* = 8.3 × 10^−6^), and the enrichment of shorter paths became stronger as the stringency of the covariation threshold increased (Figure S5b). Among covarying metabolites, 21.8% have a path length of 1 or 2. In contrast, among uncorrelated metabolites, only 13.4% have a path length of 1 or 2 (Figure S5c). This analysis suggests that pairs of metabolites that are more closely linked in the KEGG metabolic network (Krumsiek et al. 2011), which captures biochemical relationships, are more likely to exhibit covariance across samples, a statistical relationship.

To identify nodes enriched along the paths connecting metabolite pairs we first identified all shortest paths connecting all 8778 possible metabolite pairs of the 133 metabolites that mapped on the network. The shortest paths ranged from 1 to 5 nodes, often with many alternative paths of equal length (median number of equal paths = 13, range: 1–49,714). We then asked if any nodes were enriched among the paths between metabolites that covary specifically in the top or bottom fractions. This approach is like that of Kotze et al. (2013), a novel untargeted metabolomics correlation‐based network analysis incorporating human metabolic reconstructions, but extended to test the null hypothesis that a given node lies along the shortest paths due to its frequency among all such paths in the network (Section 2, Figure S1). The 22 metabolites among the top‐climbing covariance network that map to KEGG form 21 pairs in the KEGG network. However, no nodes were enriched along the paths connecting these 21 pairs (FDR > 0.05). In the covariance network of bottom flies, 29 of the pairs are in the KEGG network. Along KEGG paths connecting these pairs, we found over‐representation of node dme00430, the taurine and hypotaurine metabolism pathway (FDR = 2.63 × 10^−5^). In KEGG, node dme00430 connects taurocyamine to arabitol/xylitol, NAD, alanine, cholesteryl sulfate and hypoxanthine, which together are five of the 29 pairs (17.2%) of metabolites that covary among the poor‐climbing flies (Figure 5c). dme00430 is among the shortest paths for only 46 (0.52%) metabolite pairs in the entire KEGG network, which makes its representation among five pairs in the covariance network of the poorest climbing flies highly significant (Fisher's exact test, FDR = 2.0 × 10^−8^).

This enrichment analysis considers nodes that connect covarying metabolites, where the observed covariance is a property of metabolite pairs and not of all metabolites in the network, many of which will not covary (Kotze et al. 2013; Krumsiek et al. 2011). Once the metabolite pairs are mapped to KEGG, the null set becomes all nodes among all paths of equally short length that connect the metabolites. The bottom fraction network contains 31 metabolites in KEGG, and many paths that connect to dme00430. We assume that the enrichment of dme00430 is due to *metabolite connectivity* in this network rather than which metabolites are simply present in the bottom network. We test this assumption by asking if the identity of the 31 metabolites in the bottom network, and not specifically those that covary, could be responsible for the enrichment. To do so, we randomized the covarying pairs among these 31 metabolites, while maintaining the original degree distribution, and measured the number of randomized metabolite pairs that had the dme00430 node among their shortest paths (Section 2). If the identity of metabolites in the bottom network alone enriches dme00430, then we expect to see five such paths—the number of paths observed in the real network—appear often among the permutations. In 10,000 permutations, we saw a mean of only 0.65 such paths (empirical *p* = 0.0075, Figure S7). This result suggests that we have identified pathways that are enriched with respect to the pairs of metabolites that are correlated with one another.

## 3 Discussion

Here we show that, among isogenic flies of the same age, climbing behavior is an early‐age biomarker of stress sensitivity and mortality. Current climbing behavior predicted future mortality as early as Week 2.5 (Figure 2), and the difference in climbing velocity between flies in the top and bottom fractions persisted for days after the assay (Figure 2). This is particularly notable, given that almost no flies have died by 2.5 weeks, suggesting that climbing reveals latent, non‐genetic variation in the risk of mortality weeks or months in the future. Thus, this simple assay could serve as a surrogate biomarker, offering a powerful tool to screen for geroprotective interventions in flies (LeBrasseur 2024).

The climbing assay also allowed us to identify metabolites in the first third of lifespan that associate with climbing behavior and thus with future survival, pointing to possible mechanisms that underlie latent variation in aging. Our results do not tell us whether these metabolites, or their covariances, are a cause or consequence of the variation in climbing behavior or the associated mortality risk. However, we highlight two specific observations here, where potential causal associations between our metabolite findings and lifespan are indicated by previous studies. First, low levels of α‐ketoglutarate associate with poor climbing and higher mortality (Figure 4). Reflecting the potential causality of this association, supplemental α‐ketoglutarate can increase fly climbing and extend lifespan (Su et al. 2019). Second, metabolites whose abundance associated with climbing were enriched for the pantothenate biosynthesis module. A causal relationship is suggested by the reduced climbing and shortened lifespan of pantothenate kinase mutants, effects that are partially rescued by pharmacologically supplementing downstream metabolism (Rana et al. 2010).

We explored the use of metabolite covariance to gain insights into non‐genetic variation in aging. Previous studies in *Drosophila* found that the strength of correlations among metabolites declines with age and under dietary conditions that increase mortality (Laye et al. 2015; Lyu et al. 2021). The cause of reduced covariance is not known, but it may reflect accumulating stochastic variation, like the noise that accumulates in the transcriptome with age (Perez‐Gomez et al. 2020). In apparent contradiction to this theory, our network analysis found a more extensive set of climbing‐rank dependent covarying metabolites in the metabolome of poor‐climbing flies (Figure 5), suggesting that an overall reduction in metabolite covariance is not associated with mortality risk. We suspect that the covarying metabolites in flies of the bottom and top fraction may reflect differences in metabolism that are not detected by analysis of metabolite abundance alone and may not be realized without a way to detect those flies that are more or less likely to die.

Using a novel enrichment analysis, we identify KEGG node dme00430 enriched among the covariant metabolites in poor‐climbing flies. This node is comprised of several metabolites and enzymes and connects several components of metabolism (Kanehisa et al. 2021). We do not resolve a role for any of these entities in shaping the metabolome of poor‐climbing flies. Instead, we note that, in *Drosophila*, transsulfuration, a highly related pathway where cystine is irreversibly metabolized to taurine, is associated with longevity and the response to dietary restriction (Kabil et al. 2011; Parkhitko et al. 2016, 2019).

There are several limitations to this study worth considering. First, the fractionation procedure separated the population into eight cohorts (12.5%). This level of resolution was sufficient to identify fractions differing in mortality and to provide numerous flies for demographic and metabolomic analysis. Yet, additional fractionation may resolve an even greater degree of latent variation for future mortality risk. Second, the fractionation procedure was conducted around 11 a.m. to 1 p.m., where 9 a.m. marks the beginning of the 12 h daily light cycle in our lab. This corresponds to a period of intermediate climbing activity in the typical circadian cycle of *Drosophila* (Dubowy and Sehgal 2017), and we anticipate that fractionation earlier or later in the day may lead to different outcomes. Third, we performed these experiments with females from the common lab strain *Canton‐S*, and we do not know the degree to which the association between early‐age climbing ability and metabolomic profiles and later survival is conserved among similar fractions of male flies or from other genotypes.

Two other considerations are worth mentioning including the potential influence of genetic variation and the microbiome. Here we assume that there is little to no genetic variation in the Canton S inbred strain. In several mutation accumulation experiments, the variance in *Drosophila* longevity explained by both accumulated standing variation or mutation (*V*_M_), relative to *V*_E_, the so‐called mutational heritability $\left(h_{M}^{2}=V_{M}/V_{E}\right)$, was very low ($h_{M}^{2}=$ 1.36 × 10^−3^) (Houle et al. 1994; Houle et al. 1996). Thus, we think it unlikely that variation in climbing ability reflects genetic variation within the strain. In contrast to genetic variation, a role for the microbiome is more plausible. The experimental design we employ relies on partitioning flies from each climbing fraction into vials, where co‐fractioned flies remain together for the rest of their lives. Co‐housed *Drosophila* tend to share the microbiome (Wong et al. 2015), and so some portion of the variation in mortality and stress resistance that we observe post‐fractionation could be due to differences in microbiome composition. In this sense, it could be the case that “a few bad apples spoil the bunch,” or, on the other hand, perhaps there are beneficial microbes that accumulate among the best climbers. Future experiments with antibiotics, axenic flies, or individually housed flies may address this hypothesis.

## 4 Materials and Methods

### 4.1 *Drosophila* Stocks and Media

For all experiments, *Drosophila* from the Canton S stock (Bloomington Drosophila Stock Center #64349) were raised in polypropylene bottles containing ~50 mL of standard food: 5% dextrose, 3% sucrose, 6% corn meal, 2% yeast, 0.9% agar, 0.9% EtOH, 0.3% tegosept and 0.3% propionic acid and a small amount of dry yeast. Flies were maintained at ~200 eggs per bottle under a 12:12LD (light: dark) cycle at 25°C with 40–60%RH. For demography, adults that eclosed within a 3 days window were transferred to fresh bottles without supplemental dry yeast for 1 da to ensure mating, and then sexed on light CO_2_ into vials, each with ~10 mL standard food without supplemental dry yeast and 25 females each. At this point, the flies were 2–4 days post‐eclosion.

### 4.2 Fractionation by Climbing Behavior

For each fractionation experiment, flies were divided based on their behavior in climbing trials (illustrated in Figure S1B). A set of trials began when 8 or 9 vials of flies (~200 flies total) were pooled into a polystyrene tube (~33 cm long by 22 mm ID) that had removable foam plugs at both ends. Once the plugs were in place, ~28 cm of length was left for fly climbing. At several places along the length of the tube, including the midpoint (~14 cm), slots were cut onto the tube to allow a paper partition to be slid into place. Several slots were made to enable fractionation of flies, particularly later in life, when their climbing was slower than that of young flies. The “top” of the tube refers to the portion above the partition, and the “bottom” was below the partition.

A set of climbing trials involved up to three successive rounds (Figure S1B). At each trial, the tube was gently tapped onto a pad to bring the flies to the bottom, and flies were given enough time so that approximately half of them had crossed one of the slots cut into the tube, at which time the partition was slid into place and flies at the top and bottom of the tube were collected into separate vials. Then, the flies at the bottom in the first round were loaded into the tube again, and separated using the same procedure; each fraction was saved, and then a third round was done on the top and bottom fractions of that second trial. The same procedure was then applied to the flies that were in the top fraction in the first round, which had been held in a vial while the flies in the first bottom fraction were being further fractionated. The end result was to generate five fractions of flies: “top” were those that climbed to the top in all three trials, “top‐mid” were those that climbed to the top in two of three trials, “middle” had climbed to the top one out of two trials, “mid‐bottom” climbed to the top one of three rounds, and “bottom” being flies that were below the partition in all three rounds.

After fractionation, flies in each of the fractions were anesthetized on light CO_2_, and divided into new vials (mean 25.5, SD = 8.2, flies per vial). For metabolomics, after each replicate of the fractionation procedure (*n* = 11 across the study), one or two samples of five additional flies each from each fraction were flash frozen in liquid nitrogen and stored at −80°C. The flies in the new vials were returned to 25°C, 12:12LD and 40–60%RH, and censused every 2–3 days until all flies were dead or censored.

### 4.3 Survival Analysis

For survival analysis, every 2–3 days, flies in each vial were flipped into vials of fresh food without supplemental dry yeast, and the number of dead flies was recorded. Flies that either escaped, were killed during the transfer, or were stuck on the old food were censored. Survival analysis was conducted in R version 4.0.3 (R Core Team 2022) using the flexsurv package (Jackson 2016). Life tables were generated using a custom R script (courtesy of Scott Pletcher, Univ. of Michigan).

### 4.4 Climbing Velocity

The velocities of flies were measured in videos of groups of vials (batches), captured after flies were flipped into new demography vials. Flies were detected using the FreeClimber software (Spierer et al. 2021), and then we used a custom script to remove artifacts and estimate the velocities of individual flies (https://github.com/ben6uw/Harrison‐et‐al‐2025‐Climbers/blob/main/code/FollowFly_Workflow.r). Flies that did not climb were not measured. The effect of climbing fraction, age, apparatus position, and batch was estimated as fixed effects with a linear mixed model that included random effects of vial.

(3) $$\begin{array}{c} \text{velocity}=x+\beta_{1}\text{climbing group}+\beta_{2}\;\mathrm{age}+\beta_{3}\;\text{climbing group}\times \mathrm{age}\\ \;+\beta_{4}\;\text{position}+\beta_{5}\;\text{batch}+\left(1|\text{vial}\right)+\varepsilon \end{array}$$

### 4.5 Bang Assay

To test for bang sensitivity, flies were raised as described above. At Week 4, nine fractionations were done as before. For simplicity, we retained only the top and bottom fractions for further experiments. Following fractionation, flies were immediately assayed for bang sensitivity. For the bang assay, flies from each climbing fraction (*n* = 20–25 flies per vial) were transferred to vials without food and shaken (vortexed) for 90s. To avoid the effects of heat, shaking was done by cycling vials over three vortex mixers at 30s each, allowing the mixers to cool between cycles. As negative controls, survival was assayed in four vials per climbing fraction, which were not shaken. Flies were returned to vials with food and mortality was recorded 24 h later. For analysis, the proportion surviving per vial (1≥y≥0) was transformed to ($1>y^{\prime }>0$) by:

(4) $$y^{\prime }=\frac{y\left(n-1\right)+0.5}{n}$$

where n is the number of flies per vial. Because the proportion $y^{\prime }$ is bounded, effectively a beta distribution, we fit the effects of climbing fraction, treatment, and their interaction on $y^{\prime }$ by beta regression with the betareg package (Grün et al. 2016).

### 4.6 Metabolite Extraction and LC–MS Analysis

Metabolites were extracted from 72 samples in three batches by homogenizing frozen flies in 1.5 mL microfuge tubes each with one 5 mm diameter zirconium oxide bead (Thermo Fisher, Waltham, MA) for 2 min at 4°C and 30 Hz in a Tissuelyser II (Qiagen, Hilden, Germany). Samples were then vortexed for 10 s in 1 mL of room temperature 8:2 (v:v) HPLC grade methanol: water, incubated at −20°C for 30 min, sonicated in an ice bath for 10 min, and then centrifuged at 18,400 rcf at 4°C for 15 min. 600 μL of supernatant was transferred to a new 1.5 mL tube, dried in a vacuum centrifuge, and the pellets were stored at −80°C until suspension for LC–MS.

Targeted LC–MS analysis was performed on a duplex‐LC–MS composed of two parallel Shimadzu UPLC pumps, a temperature‐controlled auto‐sampler and an AB Sciex 6500+ Triple Quadrupole MS (Meador et al. 2020). Each sample was injected twice on two identical columns (Waters XBridge BEH Amide XP) performing separations in hydrophilic interaction liquid chromatography mode. One column performed separation and MS data acquisition in ESI+ ionization mode and the other in ESI‐ mode. Each chromatography separation was 18 min. The LC–MS ran with AB Sciex Analyst 1.6.3 software and MS peaks were integrated using AB Sciex MultiQuant 3.0.3 software. Two sets of quality control (QC) samples were injected for every ten study samples. QC(I) was a pooled human serum sample used to monitor system performance and the other, QC(S), was pooled study samples and was used to monitor data reproducibility.

### 4.7 Metabolome Data Analysis

The LC–MS peak areas for 160 targeted metabolites in 72 biological samples were log normalized and mean‐centered by sample. Effects of the three metabolite extraction batches were removed using the ComBat function in the sva package (Leek et al. 2012). Effects of LC–MS sample run order were removed by linear regression of each metabolite over run order. An outlier sample was identified by Mahalanobis distance [across PC_1–4_], of > 4 SD from the mean distance among all samples, and was removed. Principal component analysis was performed on data scaled by metabolite. Significant PCs were identified by the Tracy–Widom test in the AssocTest package (Wang et al. 2020) at *α* = 0.01.

For ordinal logistic regression on individual metabolites, climbing fractions (*j*) were ordered as “top” > “mid‐top” > “middle” > “mid‐bottom” > “bottom,” and the cumulative probability of the *i*th fly sample being in the *j*th fraction or below was fit using the clmm function of the ordinal package (Christensen 2019).

(5) $$\begin{array}{c} \text{logit}\left(P\left(Y_{i}\leq j\right)\right)=\theta_{j}-\beta_{1}\;{\mathrm{age}}_{i}-\beta_{2}\;{\text{metabolite}}_{i}\\ \;-\beta_{3}\;{\mathrm{age}}_{i}\times {\text{metabolite}}_{i}-\left(1|\text{replicate}\right) \end{array}$$

where *θ* is the *j*th threshold value, *β*_1_ is the effect of age (categorical for Week 4 or Week 6), *β*_2_ is the main effect of a metabolite, *β*_3_ is the metabolite by age effect, and (1|replicate) is the random effect of the replicate fractionation procedures. The significance of *β*_2_ or *β*_3_ was assessed by ANOVA of models with and without the metabolite or the metabolite by age interaction and the null model which includes only *β*_1_.

To identify metabolite pairs whose covariation is associated with climbing, we performed ordinal regression as above for all pairwise combinations of the *i*th and *k*th metabolite. First, the main effects of climbing fraction and age on each metabolite were removed by least squares regression.

(6) $$\text{metabolite}=x+\beta_{1}\text{climbing group}+\beta_{2}\;\mathrm{age}+\beta_{3}\;\text{climbing group}\times \mathrm{age}+\varepsilon$$

We then regressed residuals of all pairs *i* and *k* and tested for models where the interaction term *β*_3_ improved the model, when compared to the model without it, using ANOVA and FDR correction (Benjamini and Hochberg 1995).

(7) $$\begin{array}{c} \text{logit}\left(P\left(Y_{i}\leq j\right)\right)=\theta_{j}-\beta_{1}{\text{metabolite}}_{i}-\beta_{2}\;{\text{metabolite}}_{k}\\ \;-\beta_{3}\;{\text{metabolite}}_{i}\times {\text{metabolite}}_{k} \end{array}$$

### 4.8 Covariance and Network Analysis

Pairwise metabolite covariance was measured by Pearson's correlation. For KEGG network analysis, we used the buildGraphFromKEGGREST function from the FELLA package (Picart‐Armada et al. 2018) to build an undirected and unweighted network from the *D. melanogaster* KEGG database (version 108, www.genome.jp/kegg/docs/upd_all.html). The nodes along the shortest paths between all 8778 metabolite pairs were extracted from the network using the igraph package (Csardi and Nepusz 2006). We used Fisher's exact test to compare the expected and observed frequency of a given node among all nodes in all shortest paths between each metabolite pair, using the frequency of each node among paths linking all 8778 possible pairs as the null expectation.

## Author Contributions

All authors helped conceive and design the experiments. Y.S., T.N., H.S. D.D., and B.R.H. collected data and contributed to data analysis. B.R.H. analyzed data and wrote the paper. D.R. edited the manuscript. D.E.L.P. analyzed data and helped write the manuscript. All authors reviewed and approved the final manuscript.

## Conflicts of Interest

The authors declare no conflicts of interest.

## Supporting information


**Appendix S1:** acel70299‐sup‐0001‐AppendixS1.pdf.

## Data Availability

All data and code will be publicly available at https://github.com/ben6uw, as of the date of publication.
