# Supplementary material for: Early‐Life Climbing Stratifies the Metabolome and Mortality Risk in Genetically Identical Flies
Source: Aging Cell. 2025 Nov 26;25(1):e70299. doi: 10.1111/acel.70299 (PMC12740087; doi:10.1111/acel.70299)
Supplement: Supplementary file 1 — Appendix S1: acel70299‐sup‐0001‐AppendixS1.pdf. [file ACEL-25-e70299-s001.pdf]

Supplemental Material

Table of Contents

1. **Supplemental Figure S1.** Fractionation based on mid-life climbing behavior
2. **Supplemental Figure S2.** Age-dependent hazard associated with climbing fractions
3. **Supplemental Figure S3.** Latent variation in the multivariate metabolome
4. **Supplemental Figure S4.** Climbing-Associated Metabolite Covariation
5. **Supplemental Figure S5.** Metabolite pairs are close to each other on a metabolic network
6. **Supplemental Figure S6.** Covariance network enrichment analysis scheme
7. **Supplemental Figure S7.** Permutation testing of the enrichment of taurine and hypotaurine metabolism among the covariant metabolites in poor-climbing flies
8. **Supplementary Table S1 (Separately Online).** Summary Statistics for demography and bang assays

Supplemental Figure S1

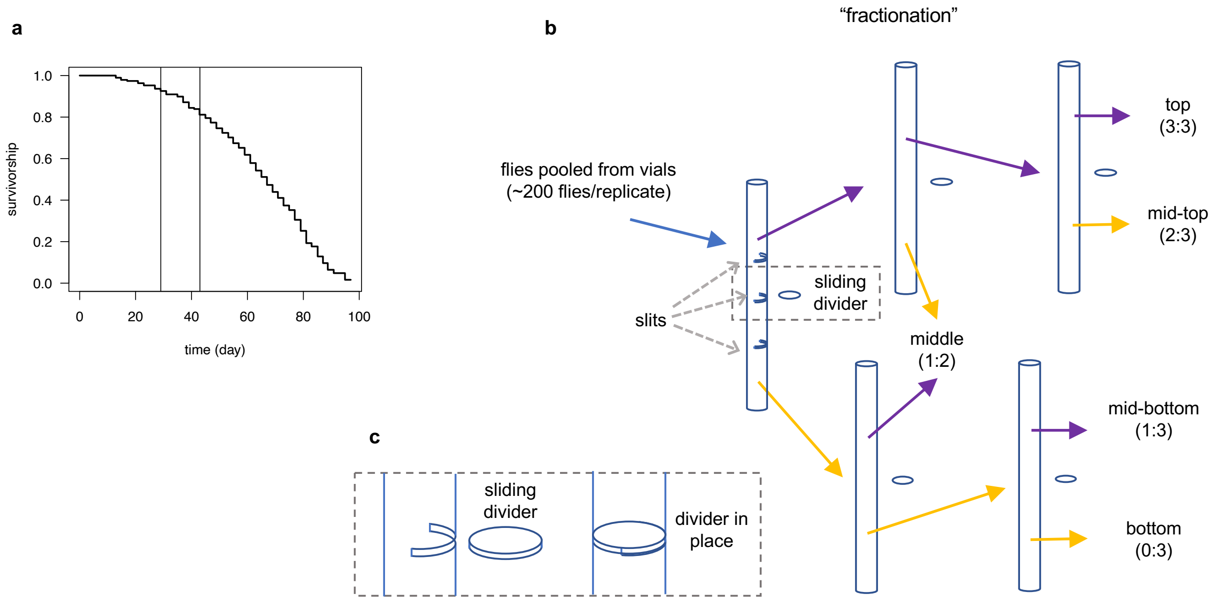

Supplemental Figure S1. Fractionation based on mid-life climbing behavior. (**a**) Survival of n=193 flies maintained in parallel to the fractionated populations, vertical lines indicate 'Week 4' (day 29) and 'Week 6' (day 43), when separate cohorts of flies were fractionated with respect to climbing behavior. (**b**) The procedure by which flies were fractioned based on their climbing behavior. Each replicate of the procedure generated five fractions of flies ('top', 'mid-top', 'middle', 'mid-bottom', 'bottom'). In each replicate, ~200 flies were pooled from fly culture vials into a polystyrene fractionation tube, with removable top and bottom seals, and a sliding divider disk that could be placed into one of several slits cut in the side of the tube (Methods). (**c**) Once approximately half of the flies had crossed one of the slits, the divider was slid into place, separating the flies on the upper and lower sections of the tube. The flies on each side were then placed in vials. These flies, from either the upper (purple arrow) or lower (yellow arrow) fraction, were then returned to the fractionation tube for subsequent fractionation. Ratios in parenthesis are the number of times that flies were in the upper portion of the tube when the divider was inserted, by the number of times they were allowed to climb.

Supplemental Figure S2

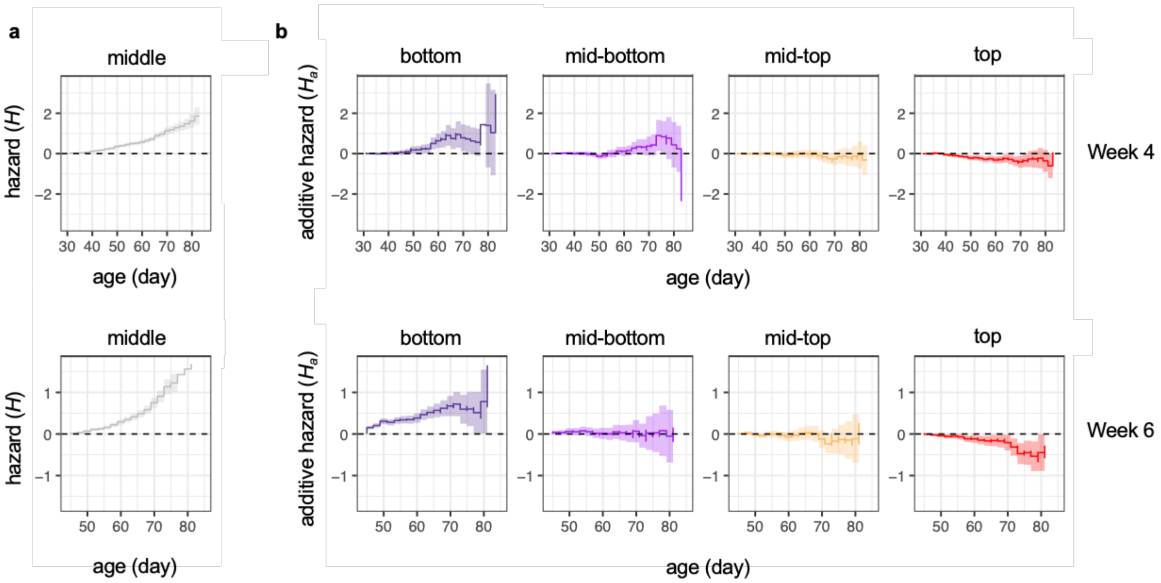

Supplemental Figure S2. Age-dependent hazard associated with climbing fractions. (**a**) The time-dependent hazard (H) fit by Aalen regression in the R package survival to the data from flies in the middle climbing group. Fits are made to data from all census intervals following the fractionation of flies at either Week 4 (day 29, top row), or Week 6 (day 43, bottom row). (**b**) Using H of the middle climbing group as a reference, the additive time-dependent hazard (H_a) of the remaining climbing groups was fit. H_a in the poorest-climbing (bottom fractions, purple), when fractioned at either Week 4 or Week 6, is greater than the reference population ($H_a > 0$, $P < 8.32 \times 10^{-4}$), whereas flies in the top-climbing fraction (top, red) from either age have reduced hazard ($H_a < 0$, $P < 0.015$).

Supplemental Figure S3

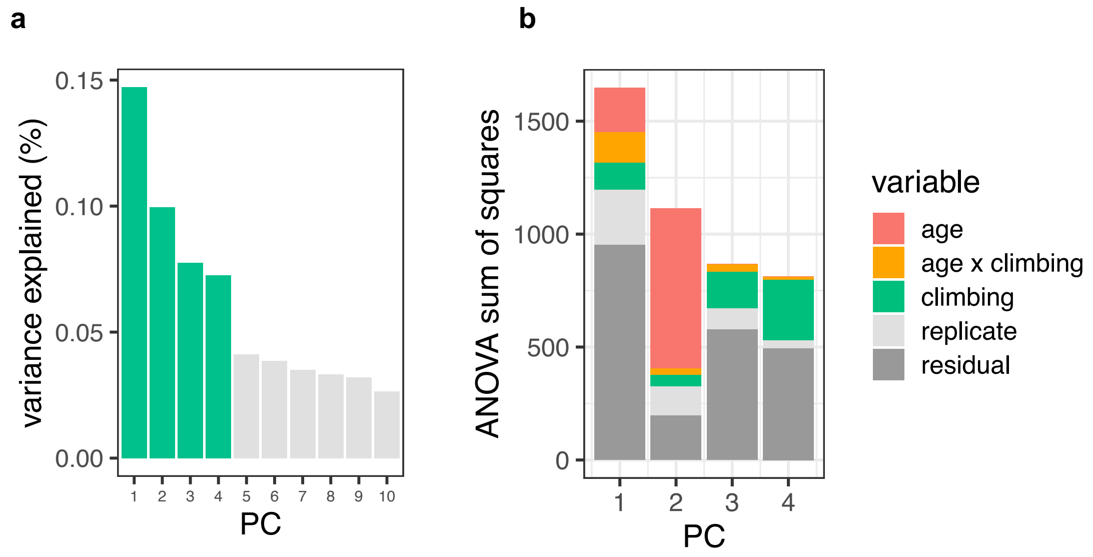

Supplemental Figure S3. Latent variation in the multivariate metabolome. (**a**) Principal components (PC) analysis of the metabolome identified the first four PCs (Tracy-Widom α<0.01, green), which explain 39.7% of the variance. (**b**) The variance (sum of squares) in each PC that could be explained by ANOVA with age (Week 4 or Week 6), climbing fraction (see text), their interaction, or the effect of fractionation replicate.

Supplemental Figure S4

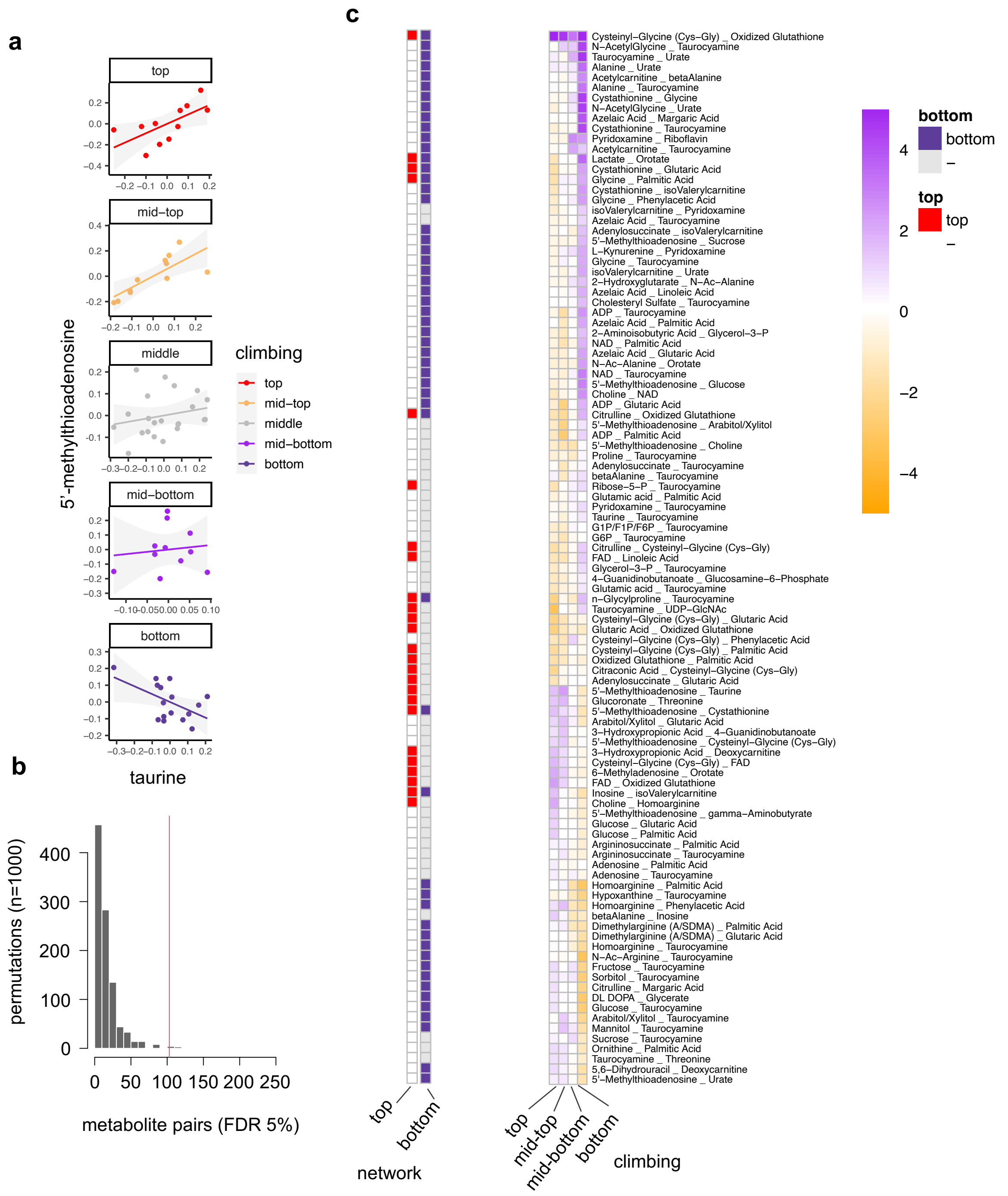

Supplemental Figure S4. Climbing-Associated Metabolite Covariation. (a) An example of metabolites whose covariance associates with ordinal climbing fraction. The abundance of 5'-methylthioadenosine and taurine among samples of each climbing fraction (n=11 to 22), lines are ordinary least square regression. (**b**) The 103 metabolite pairs whose covariance associated with ordinal climbing fractions at FDR≤0.05 in the real data (red line), compared to the number of

such pairs among each of 1000 permutations, where climbing fraction was randomized across the samples, preserving the metabolome covariation within each sample. Mean pairs from permutation 17.0 [range 0 to 244], empirical P = 0.011. (**c**) Heatmap of the 103 pairs of metabolites whose covariance associates with ordinal climbing fraction. Matrix values are the $\log_{10}$ P values, with the sign (+/-) to indicate the direction of correlation (+) or anticorrelation (-) of each metabolite pair within climbing fractions. Columns to the left of the map indicate pairs that are significant within the bottom fraction (dark blue) and top fraction (red).

Supplemental Figure S5.

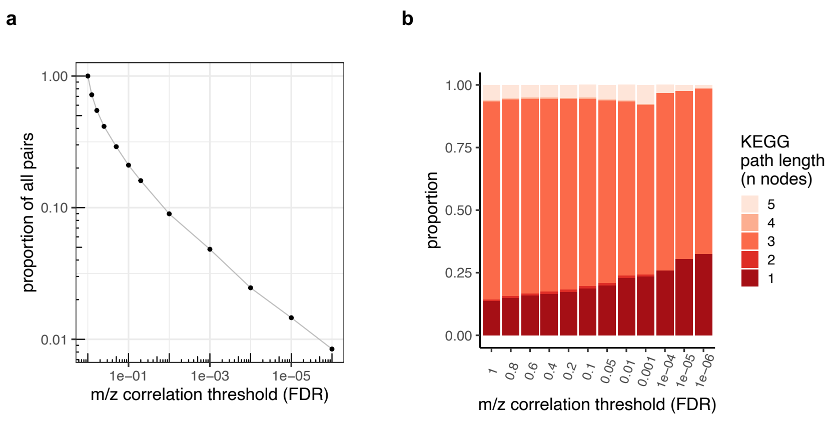

c

path length (n nodes)	pair	non-pair
1	201	999
2	13	46
3	698	6237
4	6	44
5	65	469
	983	7795

Supplemental Figure S5. Correlated metabolite pairs are close to each other on a metabolic network. 133 of 160 targeted metabolites mapped to the *Drosophila melanogaster* KEGG network. The 133 metabolites make 8778 possible pairs. (**a**) The proportion of the metabolite pairs that meet various covariation stringency (m/z correlation threshold, FDR) roughly approximates a power law. (**b**) The shortest paths connecting each metabolite pair can be expressed in as the number of nodes on the KEGG network that connect those two metabolites (KEGG path length). This length expresses how close tow metabolites lie in the KEGG network. The proportion of paths of each length, among all 8778 possible pairs, over the significance of the correlation among those pairs. When no significance threshold is applied (FDR=1, left side), the distribution of path lengths is shown. As the correlation threshold increases (from left-to-right) the metabolites meeting that threshold share disproportionately shorter path lengths (colored scale, n nodes). In other words, metabolites that are more highly correlated tend to be close to each other in the KEGG network. (**c**) A summary table of the shortest paths between all 8778 pairs. 983 of the pairs covary significantly (pair, FDR≤0.05) compared to 7795 non-pair metabolites that do not correlate (non-pair). The shortest path on the KEGG network between any two metabolites ranged from 1 to 5 nodes. Pairs at FDR≤0.05 have a slightly and significantly shorter mean path-length of 2.7 nodes compared to the path length non-pairs 2.9 edges (χ^2 P=8.3x10^{-6})

Supplemental Figure S6

v_f the focal node

q a metabolite pair

$\{p_k\}_q$ The set of k paths that connect metabolites in a pair q

$p\langle v_{i\ldots j}\rangle$ a path in KEGG, containing nodes i through j

$\mathbb{Q}_T = \binom{m}{2} =$ The set of all possible pairs of metabolites (m)

$\mathbb{Q}_r =$ The set of all pairs of metabolites that are correlated

$\mathbb{Q}_0 = \mathbb{Q}_T \notin \mathbb{Q}_r$ The set of all un-correlated pairs of metabolites

$\mathbb{N}_q$ is the union of all nodes $\{v_{i\ldots j}\}_q$ from each and all paths p in $\{p_k\}_q$.

$$\mathbb{N}_q = \cup\ (\forall p\langle v_{i\ldots j}\rangle ; p \in \{p_k\}_q,)$$

$\{\mathbb{N}_{q\ldots}\}$ The set of all $\mathbb{N}$ among the pairs of metabolites

$A = v_f \in \{\mathbb{N}_{q\ldots}\}$ = the elements of $\{\mathbb{N}_{q\ldots}\}$ that contain v_f

$B = v_f \notin \{\mathbb{N}_{q\ldots}\}$ = the elements of $\{\mathbb{N}_{q\ldots}\}$ that do not contain v_f

	$\mathbb{Q}_r$	$\mathbb{Q}_0$
A	$A \in \mathbb{Q}_r$	$A \in \mathbb{Q}_0$
B	$B \in \mathbb{Q}_r$	$B \in \mathbb{Q}_0$

Supplemental Figure S6. KEGG node enrichment among covarying metabolites. This analysis is limited to the 133 metabolites that map to the KEGG network and only considers the nodes along the shortest network paths - and not longer alternative paths - as enrichment targets. These 133 metabolites form 8,778 possible pairs ($\mathbb{Q}_T$). The shortest paths (p) connecting each pair (q) in $\mathbb{Q}_T$ were extracted from KEGG, giving a set of k paths $\{p_k\}_q$ for each of the pairs. Between the metabolites in most pairs are multiple paths of equal length ($\{p_k\}_q$) and, from each pair, $\mathbb{N}_q$ is the union of all nodes $\langle v_{i\ldots j}\rangle$ within $\{p_k\}_q$. A separate $\mathbb{N}_q$ is specific to each pair of metabolites in $\mathbb{Q}_T$, and the set of these unions is $\{\mathbb{N}_{q\ldots}\}$ and defines the total of all paths within a metabolite covariance network when it is mapped to the KEGG network.
In the top-climbing network, 21 of the pairs ($\mathbb{Q}_r$) in $\mathbb{Q}_T$ were correlated in a climbing behavior-dependent way. In the bottom-climbing network $\mathbb{Q}_r$ was composed of 29 such pairs of climbing behavior-dependent pairs (Methods). Node enrichment for the top and bottom networks was analyzed separately. In each case, the pairs in $\mathbb{Q}_T$ that are not correlated (*i.e.* in $\mathbb{Q}_r$) were denoted $\mathbb{Q}_0$.
To test for enrichment of a focal KEGG node (v_f), among paths connecting $\mathbb{Q}_r$ we determined the instances in which v_f occurred in $\{\mathbb{N}_{q\ldots}\}$ (A) versus the instances where it was absent from $\{\mathbb{N}_{q\ldots}\}$ (B). We then assemble a contingency table to denote the frequency of metabolite pairs in A and in B that are within $\mathbb{Q}_r$ and $\mathbb{Q}_0$ and used Fisher's exact test to test the null hypothesis that A and B are independent of $\mathbb{Q}_r$ and $\mathbb{Q}_0$. Thus, this procedure tests for enrichment of A in $\mathbb{Q}_r$ while controlling for the density of the network as reflected in the frequency of pairs in $\mathbb{Q}_r$ among $\mathbb{Q}_T$, and the frequency of v_f among all mapped paths in the KEGG network.

Supplemental Figure S7

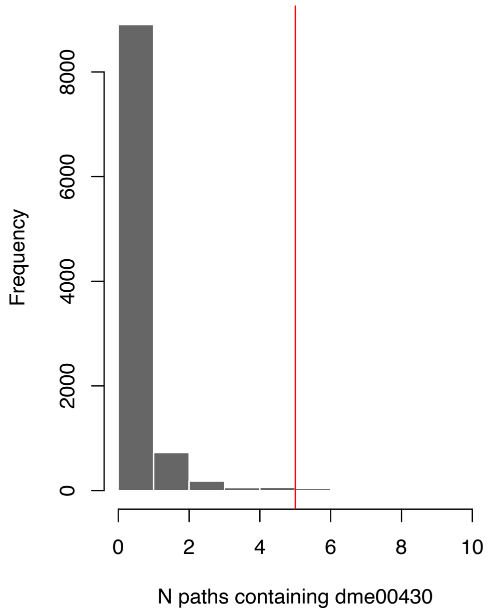

Supplemental Figure S7. Permutation testing of the enrichment of taurine and hypotaurine metabolism among the covariant metabolites in poor-climbing flies. The number of metabolite pairs in the bottom network whose paths in KEGG contain the node dme00430 (taurine and hypotaurine metabolism) in the real data (5 pairs, red line), compared to the frequency distribution among each of 1000 permutations, where the bottom covariance network was randomized, preserving the degree distribution. The mean number of pairs from permutation was 0.65 (range 0 to 10), empirical P = 0.0075.
